# Nasal floor wound coverage with an absorbable collagen matrix after revision endoscopic transsphenoidal surgery: a case report

**DOI:** 10.1097/RC9.0000000000000542

**Published:** 2026-05-15

**Authors:** Koji Suzuki, Yasuhiro Uriu, Shin Tanino, Hiroyuki Sasaki, Sawako Chiba, Kosuke Miyahara

**Affiliations:** a Department of Neurosurgery, National Hospital Organization Yokohama Medical Center, Kanagawa, Japan; b Department of Otorhinolaryngology, National Hospital Organization Yokohama Medical Center, Kanagawa, Japan; c Department of Clinical Laboratory Medicine, National Hospital Organization Yokohama Medical Center, Kanagawa, Japan

**Keywords:** case report, collagen matrix, donor-site morbidity, endoscopic skull base surgery, nasal wound healing, nasoseptal flap, revision surgery

## Abstract

**Introduction and importance:**

Absorbable collagen matrices are widely used for dural repair in neurosurgery, but their application as wound dressings in the sinonasal cavity has not been reported. Donor-site morbidity after nasoseptal flap (NSF) harvest remains a clinical challenge, particularly in revision cases with limited residual mucosa.

**Case presentation:**

An 82-year-old woman with two prior transsphenoidal surgeries (15 and 11 years prior) for a clinically nonfunctioning pituitary neuroendocrine tumor presented with progressive bitemporal hemianopsia. Imaging revealed a 37-mm recurrent sellar/suprasellar mass and near-total loss of the nasal septum. Revision surgery was pursued, given her adequate general condition and progressive optic apparatus compression. Extended NSF harvest left the nasal floor bone exposed, for which autologous donor mucosa was unavailable; an absorbable collagen matrix (DuraGen) was placed as a temporary wound dressing and secured with fibrin glue. Endoscopy on postoperative day (POD) 8 showed matrix adherence with granulation tissue. At POD 28, stable crusted coverage without infection was observed. At 3 months, the nasal floor mucosa appeared regenerated on otorhinolaryngology examination.

**Clinical discussion:**

Existing donor-site coverage strategies require harvestable autologous tissue, which was unavailable in this revision setting. The collagen matrix appeared to function as a temporary scaffold during early healing, although accelerated remucosalization could not be objectively confirmed.

**Conclusion:**

Off-label collagen matrix placement over exposed nasal floor bone was technically feasible, followed by an uncomplicated course with apparent mucosal regeneration at 3 months, and should be regarded as investigational pending prospective comparative studies.

## Introduction

The vascularized nasoseptal flap (NSF) has become a standard option for reconstruction after endoscopic endonasal skull base surgery^[^1–3^]^. However, flap harvest leaves exposed septal cartilage and bone, which can cause prolonged crusting and patient discomfort while the donor site remucosalizes^[^2,4^]^. Donor-site morbidity is particularly problematic in revision cases, where previous surgery has already depleted the septal mucosa and bony scaffold, and free mucosal grafts or mucoperiosteum are often not available in sufficient quantity. Although most donor sites eventually remucosalize, a subset of patients, particularly those who have undergone multiple prior endonasal procedures, experience persistent nasal symptoms such as crusting, epistaxis, and nasal discharge that may last months to years. Our clinical experience with such cases prompted us to explore temporary wound coverage as a strategy to support early healing and potentially reduce prolonged morbidity.

HIGHLIGHTS

- Off-label use of a collagen matrix as a nasal floor wound dressing in revision endoscopic transsphenoidal surgery.
- Septal mucosa was nearly absent after two prior transsphenoidal surgeries.
- Adherent matrix with granulation at postoperative day (POD) 8; stable, crusted coverage at POD 28.
- Nasal floor mucosa appeared regenerated by 3 months with no infectious complications.
- Single-case feasibility report; prospective comparative studies are needed.

Absorbable collagen matrices (e.g., DuraGen®) are commonly used as onlay dural substitutes and support fibroblast ingrowth and neovascularization. Their use for sinonasal wound coverage is off-label and has been rarely discussed in the context of donor-site management, particularly in revision cases with limited residual mucosa^[^5–7^]^. Shahein *et al* have described collagen matrix combined with mucoperiosteal graft as a fatless flapless reconstruction after pituitary adenoma resection[8], and Nagata *et al* have used a collagen matrix as part of a modified shoelace dural closure in extended transsphenoidal surgery[9]. However, to our knowledge, no published report has specifically addressed the use of a collagen matrix alone as a temporary wound dressing over an exposed nasal floor bone donor site after extended NSF harvest in a revision setting with near-total loss of septal mucosa. This case is therefore reported because it addresses a reconstructive gap that is increasingly relevant as the number of repeat endoscopic endonasal procedures rises.

This case has been reported in line with the SCARE 2025 criteria[10].

## Timeline

The sequence of events leading up to presentation, surgery, and postoperative follow-up is summarized in Table 1.

**Table 1:** Timeline of clinical events and endoscopic follow-up.

Time point	Event	Key finding
15 years prior	First transsphenoidal surgery (microscopic) for clinically nonfunctioning PitNET	Subtotal resection
11 years prior	Second transsphenoidal surgery (endoscopic) for tumor regrowth	Subtotal resection
Several months before admission	Progressive bilateral visual deterioration	Visual acuity: right 0.4, left 0.2; bitemporal hemianopsia
Admission (Day 0)	MRI and CT; preoperative workup	37-mm recurrent sellar/suprasellar mass; near-total loss of septal mucosa and bony septum
Day of surgery	Third transsphenoidal surgery (endoscopic) with extended NSF harvest and collagen matrix placement	Gross-total resection; Grade 3 CSF leak; multilayer reconstruction; exposed nasal floor bone covered with DuraGen®
POD 1–7	Inpatient recovery with moist nasal packing	Uneventful; visual acuity improved to 0.6 bilaterally; visual field defect resolved; no CSF leak
POD 8	Endoscopic inspection (nasal packing removal)	Matrix adherent; early granulation and neovascularization (Fig. 2C)
POD 28	Outpatient endoscopic follow-up	Dry crust covering treated area; no exposed bone, infection, or purulent discharge (Fig. 2D)
3 months postoperative	Outpatient otorhinolaryngology follow-up	Nasal floor mucosa appeared regenerated and comparable to surrounding native mucosa (no photographic documentation)

CSF, cerebrospinal fluid; NSF, nasoseptal flap; PitNET, pituitary neuroendocrine tumor; POD, postoperative day.

## Case presentation

**Figure 1.**
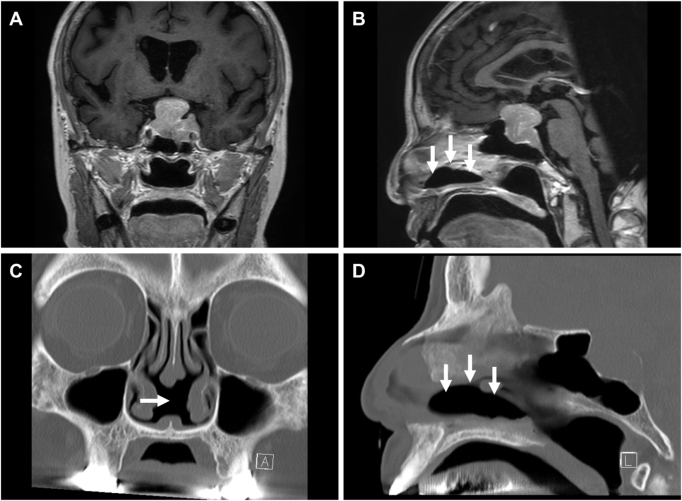
Preoperative imaging. (A) Gadolinium-enhanced T1-weighted MRI (coronal) shows a recurrent sellar/suprasellar tumor. (B) Gadolinium-enhanced T1-weighted MRI (sagittal) shows suprasellar extension and a nasal septal mucosal defect (arrows). (C and D) Coronal and sagittal CT images show marked loss of the bony nasal septum and adjacent septal mucosa (arrows).

An 82-year-old woman presented with progressive bilateral visual deterioration and bitemporal hemianopsia. Visual acuity was 0.4 in the right eye and 0.2 in the left eye. She had undergone two prior transsphenoidal surgeries for a clinically nonfunctioning pituitary neuroendocrine tumor (PitNET) 15 and 11 years before the current surgery, both resulting in subtotal resection. Magnetic resonance imaging demonstrated a 37-mm recurrent sellar/suprasellar mass with rightward deviation of the pituitary stalk (Fig. 1A and B). Computed tomography showed near-total loss of the nasal septal mucosa and bony septum related to prior surgery (Fig. 1C and D).

Despite her advanced age and the risks inherent in a third transsphenoidal procedure, revision surgery was pursued because the patient’s general condition was adequate for surgery, and the regrowing tumor was compressing the optic apparatus with progressive visual deterioration. The severely depleted donor bed was anticipated to complicate both tumor access and skull base reconstruction.

Revision endoscopic transsphenoidal surgery was performed. The tumor was soft, and gross-total resection was achieved. Because the arachnoid and sellar diaphragm were largely deficient, a high-flow cerebrospinal fluid (CSF) leak occurred (Grade 3)[11].

**Figure 2.**
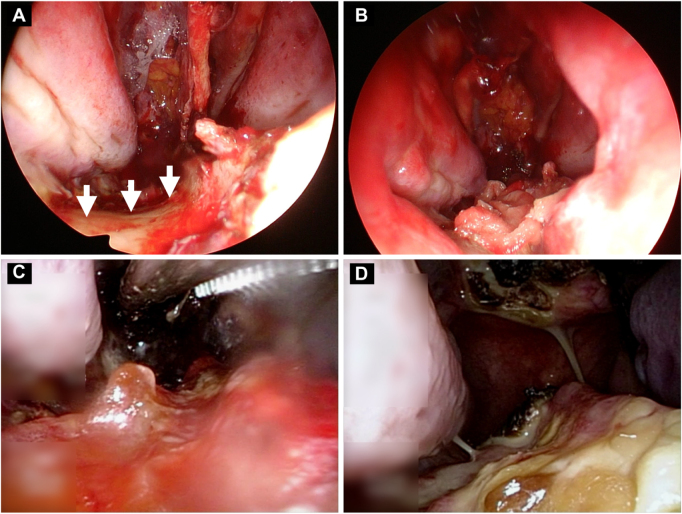
Endoscopic views. (A) Intraoperative view after extended flap harvest; arrows indicate exposed nasal floor bone. (B) Placement of the absorbable collagen matrix (DuraGen®) on the exposed bone. (C) POD 8 endoscopic view showing adherent matrix with granulation tissue and neovascularization. (D) POD 28 endoscopic view showing a dry crust covering the treated area without obvious exposed bone or purulent discharge. The on-screen labels visible in panels C and D do not contain patient-identifiable information.

Multilayer skull base reconstruction was performed using autologous tissue and a vascularized NSF. Due to limited residual mucosa, flap harvest was extended to include nasal floor mucosa, leaving an area of exposed nasal floor bone (Fig. 2A). Because autologous donor mucosa for covering the exposed bone was essentially exhausted, and because we wished to avoid introducing a second distant donor site in an 82-year-old patient, we elected to place an absorbable collagen matrix as a temporary wound dressing. A 4 × 1 cm piece of absorbable collagen matrix (DuraGen®; Integra LifeSciences, Plainsboro, NJ, USA) was trimmed, moistened with saline, placed directly on the exposed bone, and secured with fibrin glue (Beriplast®; CSL Behring, Marburg, Germany) (Fig. 2B). Moist gauze packing was left in the nasal cavity for 1 week as part of routine postoperative care to support a humid environment.

The postoperative course was uneventful. Visual acuity improved to 0.6 bilaterally, and the visual field defect resolved. The patient was discharged in stable condition. Endoscopic examination on postoperative day (POD) 8 showed that the collagen matrix remained adherent, with early granulation tissue and neovascularization (Fig. 2C). At POD 28, a dry crust covered the treated area; there was no obvious exposed bone, purulent discharge, or clinical evidence of infection (Fig. 2D). At 3 months postoperatively, otorhinolaryngology outpatient examination demonstrated that the nasal floor mucosa had regenerated and appeared comparable to the surrounding native mucosa; no photographic documentation was obtained at this visit. The patient reported no nasal discharge, epistaxis, nasal obstruction, foul odor, or olfactory disturbance at any point after packing removal on POD 7.

## Patient’s perspective

The patient reported that immediately after surgery, she experienced nasal discharge and minor epistaxis, similar to what is expected after routine transsphenoidal surgery. After the removal of the nasal packing on POD 7, these symptoms resolved. She subsequently had no nasal discharge, epistaxis, nasal obstruction, foul odor, or olfactory disturbance and remained asymptomatic throughout the follow-up period.

## Clinical discussion

In NSF donor sites, exposed cartilage or bone typically remucosalizes from the surrounding mucosal edges and may require 10–12 weeks. During this interval, crusting and the need for repeated debridement are common[2]. However, a subset of patients, particularly after revision surgery with extensive mucosal loss, may experience persistent nasal symptoms such as crusting, epistaxis, and nasal discharge that last months to years and significantly impair quality of life. Our clinical experience with such cases motivated the present approach of applying a collagen matrix as a temporary wound dressing to support early healing in an especially challenging donor site.

Collagen matrices are designed to act as temporary extracellular scaffolds that support cellular migration and tissue remodeling. In neurosurgery, onlay collagen matrices have been used safely for dural repair with acceptable long-term outcomes^[^5–7^]^.

In the present case, an absorbable collagen matrix was applied off-label to exposed nasal floor bone after extended flap harvest in a revision setting with limited residual mucosa. Early endoscopy demonstrated adherence of the matrix and granulation tissue formation. At 4 weeks, the appearance was dominated by crusting, which can represent part of normal secondary healing in the sinonasal cavity rather than definitive proof of complete epithelialization. At 3 months, the nasal floor mucosa appeared regenerated on clinical examination; however, histological confirmation was not performed. Therefore, we interpret this single-case observation primarily as a feasibility description of temporary wound coverage rather than evidence of accelerated remucosalization, and we have intentionally avoided drawing generalizable conclusions from a single patient.

The sinonasal cavity differs from a closed, continuously wet surgical cavity used for dural repair. To reduce desiccation, the matrix was moistened at placement and supported by moist nasal packing during the first postoperative week. Potential risks include infection and foreign-body reaction; in this case, no infectious complications were observed.

Numerous vascularized and free-tissue reconstructive strategies have been developed for endoscopic skull base surgery, as comprehensively reviewed in the International Consensus statement on Allergy and Rhinology: Endoscopic Skull-Base Surgery (ICAR: ESBS)[12]. When the standard NSF is unavailable or insufficient, alternative vascularized flaps include the lateral nasal wall flap[13], the superiorly based middle turbinate flap[4], the upper-tongue nasopharyngeal flap[14], and various extended NSF modifications incorporating inferior turbinate or lateral nasal wall mucosa[3]. However, these alternatives are designed primarily for dural or skull base defect reconstruction and require harvestable vascularized tissue, which was not available in sufficient quantity in our revision patient. For the separate problem of donor-site coverage, free middle turbinate mucosal grafting, as described by Kimple *et al*, provides autologous epithelial coverage and has been shown to reduce crusting[2], but requires harvestable turbinate mucosa. Mucoperiosteal flaps and combined collagen matrix–mucoperiosteum constructs, as reported by Shahein *et al*[8], also rely on residual autologous mucosa from the nasal floor or septum. Modified shoelace dural closure with collagen matrix, as described by Nagata *et al*[9], is aimed primarily at dural reconstruction rather than donor-site coverage. In contrast, the technique described here uses a collagen matrix alone, without any autologous mucosal graft, specifically as a temporary wound dressing over an exposed nasal floor bone donor site in a setting where autologous options are effectively exhausted. The present report therefore extends the discussion around donor-site management by documenting the feasibility of matrix-only coverage in such revision anatomy; it does not propose this approach as a first-line replacement for established mucosal grafting techniques when adequate donor tissue is available.

### Strengths and limitations

Strengths of this report include a clearly documented, reproducible operative technique; endoscopic images at two defined postoperative time points; clinical follow-up through 3 months with mucosal assessment by otorhinolaryngology; and transparent reporting in line with SCARE 2025. Limitations include the single-case design without a control; the absence of objective measures of healing, such as standardized endoscopic scoring (e.g., Lund–Kennedy score), histological confirmation of epithelialization, or validated patient-reported outcome measures (e.g., SNOT-22); the lack of photographic documentation at the 3-month visit; and a follow-up period that may be insufficient to detect late complications. Therefore, any claim of accelerated remucosalization remains speculative, and the observations should be interpreted as hypothesis-generating. Future comparative studies using serial Lund–Kennedy endoscopic scores, time to complete remucosalization, and validated sinonasal quality-of-life questionnaires at defined postoperative intervals are required to determine whether collagen matrix placement reduces donor-site morbidity or accelerates remucosalization. Prospective registries of revision endoscopic endonasal cases may be particularly suitable for testing this hypothesis, given the relatively low frequency of such anatomy.

## Conclusion

Off-label placement of an absorbable collagen matrix over exposed nasal floor bone after revision endoscopic transsphenoidal surgery was feasible and was followed by an uncomplicated early healing course. Three take-home messages follow from this single-case experience: (1) a collagen matrix can be used as a temporary wound dressing over a nasal floor bone donor site when autologous mucosa is exhausted; (2) early endoscopic findings (matrix adherence, granulation, absence of infection, and apparent mucosal regeneration at 3 months) support short-term safety but do not prove accelerated remucosalization in the absence of objective measures; and (3) the technique should, at present, be regarded as investigational and reserved for selected revision cases, with wider adoption contingent on prospective comparative data using objective healing endpoints.

## Data Availability

Not applicable.
